# Enhancing drought stress mitigation in faba bean through natural biostimulants derived from banana and prickly pear peels

**DOI:** 10.1186/s12870-026-08730-4

**Published:** 2026-04-28

**Authors:** Samah M. Megahed, Amany Gaber Mohamed, Eman M. M. Eldebawy, Manal M. Abdel-Rahman, Masarrat M. Migahid, Roufaida M. Elghobashy

**Affiliations:** 1 Biology and Geology Department, Faculty of Education, Alexandria University, Alexandria, Egypt; 2 Botany and Microbiology Department, Faculty of Science, Damanhour University, Elbehira, Egypt; 3 Plant Pathology Department (Genetics branch), Faculty of Agriculture, Damanhour University, Elbehira, Egypt

**Keywords:** Climate resilience, Crop productivity, Gene expression, Growth promotion, Stress tolerance, Sustainable agriculture

## Abstract

**Background:**

The use of biostimulants in agriculture has shown fabulous potential in combating climate change induced stresses such as drought. Improving agricultural productivity requires sustainable drought stress management. This study investigates the potential of *Musa paradisiaca* L. (banana) and O*puntia ficus-indica* (L.) Mill. (prickly pear) peels as natural biostimulants to alleviate drought stress in *Vicia faba* L. (faba bean). A greenhouse experiment was conducted using peel powder treatments (5 g/pot) under different field capacity levels to evaluate germination parameters, plant biomass and water content. Leaf ultrastructure and stress-related gene expression were also evaluated. In addition, the physicochemical characterization of peel powders, including soluble protein, soluble sugars, total phenolics and flavonoids, was performed.

**Results:**

The physicochemical characterization of banana and prickly pear peel powders revealed the presence of considerable amounts of phenolics, flavonoids, sugars, and proteins, with higher phenolic and flavonoid contents detected in *Musa paradisiaca* peel powder. The application of both peel extracts, particularly *Musa paradisiaca* L., significantly improved shoot and root length, plant biomass, and water content compared to untreated control plants. Under representative drought stress, most growth parameters and water content of the studied plants were higher in *Musa paradisiaca* L.-treated plants compared to their respective control, whereas *Opuntia ficus-indica* (L.) Mill. treatment showed comparatively lower values. TEM observations revealed improved leaf ultrastructure in treated plants under drought conditions. While qRT-PCR analysis indicated upregulation of stress-related genes. The transcript level of ascorbate peroxidase was significantly higher in plant treated with *Musa paradisiaca* L. than in plant treated with O*puntia ficus-indica* (L.) Mill. when exposed to moderate drought (40%FC), suggesting a potential involvement of APX in antioxidant-related stress responses under drought conditions.

**Conclusion:**

These results suggest that natural peel powders could be a viable strategy for promoting sustainable agriculture and improving plant performance under drought. Overall, modulation of stress-related gene expression may contribute to improved drought responses in faba bean. These effects appear to be associated with improved water status, preservation of cellular ultrastructure, and regulation of stress-responsive pathways.

## Introduction

Drought is one of the most destructive abiotic stresses threatening global food security, limiting plant performance at physiological, biochemical, and molecular levels [1, 2]. By 2050, it is expected to affect over half of the world’s arable land, severely reducing agricultural productivity [3]. Biostimulants are substances that, when applied to plants, enhance growth, nutrient uptake, and tolerance to abiotic stresses, without being fertilizers or pesticides, acting through diverse physiological and molecular mechanisms [4]. They can modulate the expression of genes involved in metabolic, developmental, and physiological processes, including antioxidant enzymes, thereby enhancing plant tolerance to water deficit conditions [5].

The faba bean (*Vicia faba* L. 2n = 12) is a key legume crop valued for its high protein content (20–30%) in both human and animal diets [6–8]. However, most cultivars are highly sensitive to abiotic stresses, and the lack of sufficient genetic tolerance contributes to yield instability. Improving both yield and stress tolerance remains a major target in faba bean breeding [9]. Fruit processing generates large amounts of by-products, many of which contain higher levels of phytochemicals and nutrients than the edible pulp [10]. Exploiting these residues as natural biostimulants or soil amendments offers a low-cost, eco-friendly strategy for enhancing crop performance. The peel of banana (*Musa paradisiaca* L.) and prickly pear (*Opuntia ficus-indica* (L.) Mill.) are particularly plentiful in bioactive compounds.

Banana peels contain phytochemicals and antioxidants including phenolics, flavonoids, vitamins, β-carotene, potassium, calcium, and magnesium, in addition to proteins, dietary fiber, amino acids (notably tryptophan), and polyunsaturated fatty acids [11]. These constituents make banana peel a promising plant growth-promoting material suitable for soil or foliar application [12, 13]. Indeed, banana peel soil supplementation has been shown to reduce the adverse effects of water stress [14]. Globally, banana consumption generates nearly 36 million tons of peel waste annually [15, 16], highlighting its potential as a sustainable agricultural input.

*Opuntia ficus-indica*, widely recognized as the prickly pear, represents one of the most economically important species within the Opuntia genus owing to its notable health benefits. This plant is rich in bioactive constituents such as betalains, phenolics, sterols, dietary fibers, and essential vitamins, especially vitamin C [17, 18]. The processing of prickly pear fruits generates considerable amounts of by-products such as peels and seeds, which still contain significant levels of nutrients and bioactive compounds [19].

Instead of being undervalued as livestock feed or discarded, these by-products are increasingly recognized as a promising resource for sustainable agricultural practices. In particular, the phenolic-rich peels have attracted attention for their potential role as natural biostimulants, contributing to stress tolerance and improved crop performance under challenging environmental conditions [20–22]. However, the high moisture content of the peel (75–85%) makes it highly perishable, thus necessitating further processing for safe and efficient utilization [19, 23].

Plants exhibit various adaptive strategies to cope with abiotic stresses such as drought, and one of the most important mechanisms involves the activation of antioxidant enzymes [24–28]. Hernández et al. [29] reported that an increase in the expression of genes encoding antioxidant enzymes enhances plant tolerance to drought stress by protecting cells against oxidative damage. Several studies have therefore investigated the expression of such genes to better understand the activity of antioxidant enzymes under stress conditions. For example [30], observed higher expression of ascorbate peroxidase (APX), SOD, and CAT genes, together with increased enzyme activity, in drought-resistant barley genotypes under severe drought compared to susceptible ones. Similarly [31], found that peroxidase (POX), cytosolic ascorbate peroxidase (cytAPX), and CAT genes were more highly expressed in a tolerant canola cultivar. In contrast [32], demonstrated that drought-sensitive wheat cultivars exhibit low expression levels of APX, DHAR, and GR isoforms, reflecting inadequate protection against oxidative stress under water deficit conditions. Overall, these findings highlight that high expression levels of antioxidant enzyme genes improve plant protection against reactive oxygen species (ROS) under drought and other relevant abiotic stresses.

Although banana and prickly pear peels have been reported as organic amendments or biostimulants in various crops, their combined physiological, ultrastructural, and molecular effects on faba bean (*V. faba* L.) under controlled drought stress remain largely unexplored. Therefore, this study aims to evaluate the efficacy of *Musa paradisiaca* and *Opuntia ficus-indica* peels as natural biostimulants to mitigate drought stress in *V. faba* plants. Specifically, it investigates their effects on physiological, biochemical, ultrastructural, and molecular responses under varying water availability conditions. By integrating these natural resources into crop management practices, this study aims to promote sustainable farming strategies that enhance both productivity and resilience in response to changing climatic conditions.

## Materials and methods

### Preparation of plant materials

Fresh peels of *Musa paradisiaca* L. (banana) and *Opuntia ficus-indica* (L.) Mill. (prickly pear) were collected from a local market, washed thoroughly with clean water to remove any dirt and contaminants that were adhering. then cut it into small pieces to facilitate drying and grinding. The peels were first air-dried at room temperature for several days with occasional turning to prevent mold growth, followed by oven-drying at 40 °C for 5 days. Fully dried peels were ground into a fine powder and stored in airtight containers until use. Seeds of *V. faba* L. (faba bean) were obtained from the International Research Center, El-Dokki, Giza, Egypt.

### Biochemical characterization of peel powders

Basic biochemical analyses were conducted to determine the soluble protein, soluble sugars, total phenolic, and total flavonoid contents of *Musa paradisiaca* L. (banana) and *Opuntia ficus-indica* (L.) Mill. (prickly pear) peel powders. These analyses provided insight into the major bioactive constituents of the peels, supporting their potential use as natural biostimulants. Soluble protein content was determined according to the method of Hartree [33], a modification of the Lowry assay. Absorbance was measured at 650 nm, and protein concentration was calculated using a bovine serum albumin standard curve. Soluble sugars were quantified following the phenol–sulfuric acid method described by Dubois et al. [34]. Absorbance was recorded at 490 nm, and sugar content was expressed as mg glucose equivalents per g dry weight. Total phenolic compounds were extracted with 80% methanol and determined using the modified Folin–Ciocalteu method [35, 36]. Absorbance was measured at 765 nm, and results were expressed as mg gallic acid equivalents (GAE) per g dry matter. Total flavonoid content was estimated using the aluminum chloride colorimetric method [37]. Absorbance was measured at 367 nm, and flavonoid content was expressed as µg quercetin equivalents (QE) per g dry matter. All measurements were performed in triplicate.

### Preparation of seeds for laboratory experiments

Before sowing, the uniform seeds of *V. faba* L. were surface sterilized in 3% sodium hypochlorite solution for 3–4 min and then rinsed thoroughly three times with distilled water to remove any traces of the disinfectant.

### Growth bioassay

The experiment was conducted in 2024 at the Faculty of Education, Department of Biological and Geological Sciences. A pot experiment was set up to assess the effect of banana and prickly pear peel powders on faba bean growth. The peel powder was applied at a rate of 5 g/pot, which was determined to be optimal based on preliminary greenhouse experiments and supported by previous reports showing effective growth promotion and stress alleviation in legume crops [38, 39]. This dose was sufficient to enhance growth without any observed phytotoxic effects. Treatments were arranged in a completely randomized design, with three independent pots per treatment, each pot representing one biological replicate. Ten seeds were sown per pot (16 cm diameter × 15 cm height) filled with 1000 g of clay loam soil (pH 7.7; N: 1.1 mg g⁻¹; P: 0.5 mg g⁻¹; K: 3.5 mg g⁻¹). Pots were irrigated at four different field capacity (FC) levels: 20%, 40%, 60%, and 80%. Precise drought stress was imposed by calculating the volume of water added to each pot based on daily soil–plant evapotranspiration. Each pot was weighed every 24 h, and the water lost due to evaporation and transpiration was replaced to maintain the target field capacity levels, ensuring consistent and accurate water stress conditions throughout the experiment.

The experiment was conducted under greenhouse conditions (20 ± 2 °C temperature, 75 ± 2% relative humidity, and 14/10 h light/dark photoperiod). After 33 days of growth, uniform seedlings were carefully uprooted from each treatment, washed with tap water to remove adhering soil, and gently blotted dry with filter paper. The samples were separated into roots and shoots for growth parameter measurements. Dry weight was determined by oven-drying at 60 °C until a constant weight was achieved.

### Specific analyses

#### Germination parameters

Germination and early growth traits were assessed, including shoot length (SL), root length (RL), and the shoot-to-root ratio (SL/RL). Growth parameters measurements were recorded from five randomly selected plants per pot.

#### Plant biomass and water content

Biomass measurements were recorded from five randomly selected plants per pot, separated into shoots and roots, and weighed individually to record fresh weight. The samples were then oven-dried at 60 °C until constant weight was obtained to determine dry biomass.

The water content percentage (WC%) of the shoot system was calculated using the following formula:

$$\mathrm{WC}\,\%=\left(\text{Fresh wt.}-\text{Dry wt.}\right)\ast100/\text{Fresh wt.}$$

#### Transmission Electron Microscope (TEM)

Leaf samples (second fully expanded leaf) were collected from three independent plants per treatment, each originating from a separate pot (biological replicate) [40]. Ultra-thin sections were obtained using a diamond knife mounted on an ultramicrotome (Leica EM UC6, Germany) and placed on 300-mesh copper grids. The cellular ultrastructure was examined and photographed under a transmission electron microscope (TEM) (Tokyo, Japan) at different magnifications. Representative images were captured for comparative analysis of drought-induced ultrastructural changes. Leaf sections for TEM were prepared from the second fully expanded leaf of three independent plants per treatment. A limited number of images were captured, and representative images showing consistent ultrastructural features were selected for presentation in the Results section.

#### RNA extraction, cDNA synthesis, and qRT-PCR analysis

For gene expression analysis, three independent biological replicates per treatment were used, with each replicate corresponding to leaf tissue collected from a single plant grown in a separate pot. Each qRT-PCR reaction was performed in triplicate as technical replicates. Total RNA was extracted from fresh bean leaves according to the method of [41], with minor modifications. Leaf samples were immediately frozen in liquid nitrogen and stored at − 80 °C until use. The tissues were ground in TRIzol reagent and incubated for 15 min, followed by the addition of chloroform and incubation for a further 15 min. The aqueous phase was mixed with isopropanol (1:1, v/v) and incubated at 20 °C for 45 min. After centrifugation, the RNA pellet was washed with ethanol and centrifuged again at 14,000 rpm. The pellet was finally dissolved in DEPC-treated water. Reverse transcription was carried out using the ABT H-minus kit following the manufacturer’s instructions. Each 20 µl reaction mixture contained 3 µl total RNA, 2 µl reverse transcriptase buffer, 2 µl dNTPs, 3 µl oligo (dT) primer, and 0.3 µl (1 U) reverse transcriptase. The reactions were incubated at 42 °C for 60 min, followed by heating at 70 °C for 5 min to terminate the reaction, as modified from [42]. The synthesized cDNA was stored at − 20 °C until further use.

Quantitative real-time PCR (qRT-PCR) was performed to assess the expression of three antioxidant-related genes: APX (Ascorbate Peroxidase), SOD (Superoxide Dismutase), and GR (Glutathione Reductase). These genes were selected as representative markers of antioxidant pathways, as they are widely used to indicate drought-induced oxidative stress responses in legumes [43–45]. Each cDNA sample was run in triplicate as technical replicates, and three independent biological replicates were used per treatment, with each replicate corresponding to leaf tissue collected from a single plant grown in a separate pot. Reactions were performed using gene-specific primers with SYBR Green Master Mix. The sequences of the forward and reverse primers are listed in Table 1. Relative transcript levels (RTL) were calculated using the 2^−ΔΔCt method, normalizing against the β-actin gene as an internal control [46]. Gene expression analysis was conducted only at 80% field capacity (well-watered control) and 40% field capacity (moderate drought stress), as these levels represent optimal and physiologically responsive drought conditions, respectively. Severe drought (20% FC) was excluded to avoid irreversible stress-induced damage, while 60% FC represents mild stress with limited transcriptional response. The expression levels of the target genes were normalized against the β-actin gene as internal control. Relative transcript levels (RTL) were calculated using the 2^−ΔΔCt method, where ΔCt = Ct (target gene) − Ct (reference gene), according to Kozera and Rapacz [47].

**Table 1:** Forward (F) and reverse (R) primer sequences used for qRT-PCR amplification of antioxidant-related genes (GR, APX, SOD) and the reference gene (β-actin) in *Vicia faba* L

Gene	Primer sequence	
GR	F	GATTTAGGCCAGGCGGA
	R	TCAATTTCATCTGATCATATAACAGAACCC
APX	F	GTTTCAGGCAGGCAGCA
	R	ATTCGCATTGTTCTGGGAATC
Sod	F	TGCTCGCCGAAGGACATA
	R	CCAAAGCCATTGTGAATCGAG
β-ctin	F	GTCGGTGAAGGGGACTTACA
	R	TTCATACAGCAGGCAAGCAC

### Statistical analysis

Data were expressed as mean ± standard error (SE) of three replicates. Differences among treatments and irrigation levels, as well as their interaction, were analyzed using two-way ANOVA. Mean comparisons were performed using Tukey’s honestly significant difference (HSD) test at *P* ≤ 0.01. Columns sharing the same letter indicate no significant difference between treatments.

## Results

The biochemical composition of banana (*Musa paradisiaca*) and prickly pear (*Opuntia ficus-indica*) peel powders was evaluated in terms of soluble proteins, soluble sugars, total phenolics, and total flavonoids (Table 2). Overall, Opuntia peel exhibited higher levels of soluble proteins and sugars, whereas banana peel contained higher amounts of total phenolic compounds. Total flavonoid content was relatively similar between the two peel types.

**Table 2:** Biochemical composition of *Musa paradisiaca* and *Opuntia ficus-indica* peel powders

Peel powder	Soluble protein (mg g⁻¹ DW)	Soluble sugars (mg g⁻¹ DW)	Total phenolics (mg GAE g⁻¹ DW)	Total flavonoids (µg QE g⁻¹ DW)
*Musa paradisiaca*	79.26 ± 1.44	11.67 ± 0.67	29.78 ± 0.10	9.38 ± 0.024
*Opuntia ficus-indica*	170.03 ± 1.58	48.67 ± 0.33	23.86 ± 0.13	9.176 ± 0.016

Values are expressed as mean ± SE (*n* = 3). *DW* dry weight, *GAE* gallic acid equivalents, *QE* quercetin equivalents

**Fig. 1:**
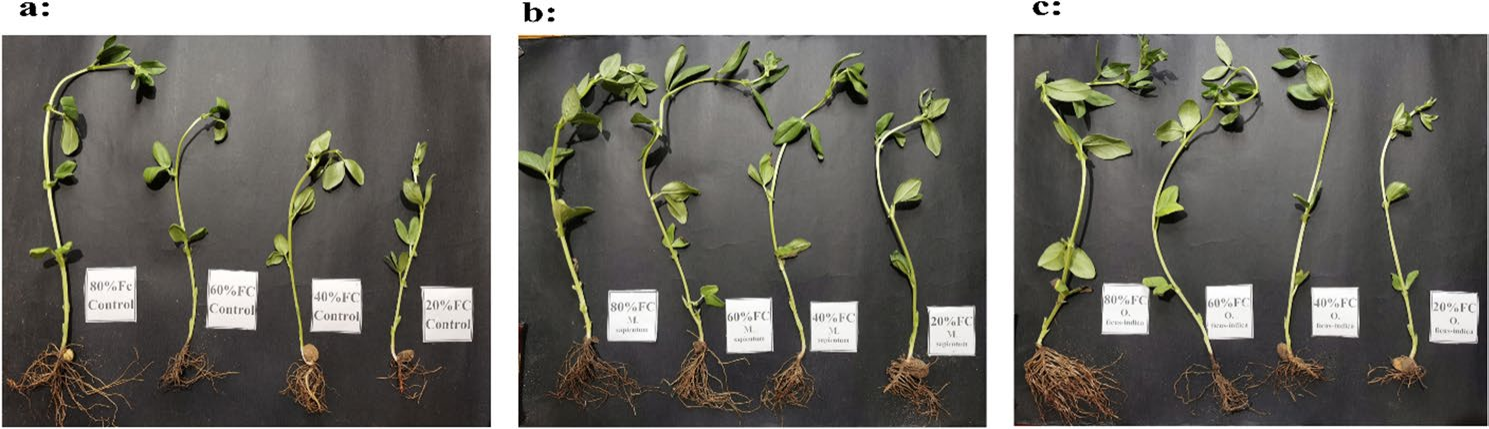
Effect of *Opuntia ficus-indica* (L.) Mill. (prickly pear) and *Musa paradisiaca* L. (banana) peel powder treatments (5 g/pot) on the growth of *Vicia faba* L. (faba bean) under different irrigation levels (20%, 40%, 60%, and 80% field capacity). **a **control, **b ***Musa paradisiaca* peel powder, **c ***Opuntia ficus-indica* peel powder

**Fig. 2:**
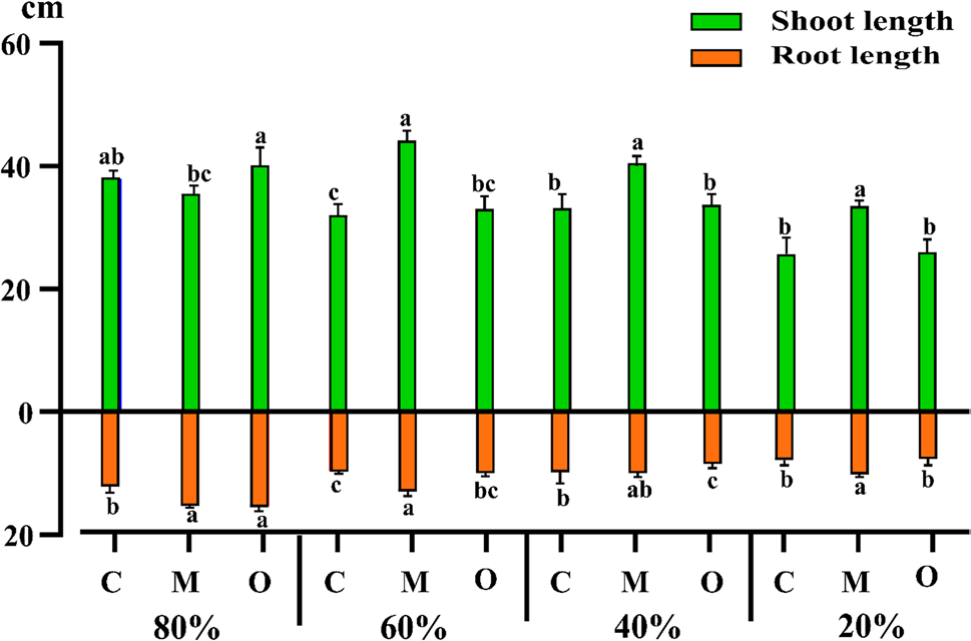
Effect of *Musa paradisiaca* L. (banana) and *Opuntia ficus-indica* (L.) Mill. (prickly pear) peel powder treatments (5 g/pot) on shoot and root length of *Vicia faba* L. (faba bean) under different irrigation levels (20%, 40%, 60%, and 80% field capacity). Bars represent mean ± SE of shoot and root length. Different letters above bars indicate significant differences among treatment × drought level combinations according to Tukey’s HSD test at *P* ≤ 0.01

Figure 1 showed significant variations in growth parameters of *Vicia faba* L. across different irrigation levels (20%, 40%, 60%, and 80% field capacity, FC). Two-way ANOVA indicated that both treatment and field capacity significantly affected shoot and root lengths. For shoot length, F-values were 11.17 for treatment and 14.3 for FC (P ≤ 0.01), with a significant interaction between treatment and FC (F = 3.8, P = 0.0028). Similarly, for root length, treatment and FC significantly affected growth (F = 10.5 and 13.8, respectively, P ≤ 0.01), with a significant interaction (F = 3.2, P = 0.005). Plants supplemented with *Musa paradisiaca* peel powder (5 g/pot) exhibited the greatest enhancement in both shoot and root lengths compared to the control across all irrigation levels. The maximum increase in shoot length (38.03% above control) was recorded at 60% FC. Under severe water stress (20% FC), both shoot and root lengths were markedly reduced; however, treated plants still attained the highest values, reaching 30.5% and 29.8% above control for shoot and root length, respectively (Fig. 2).”

**Fig. 3:**
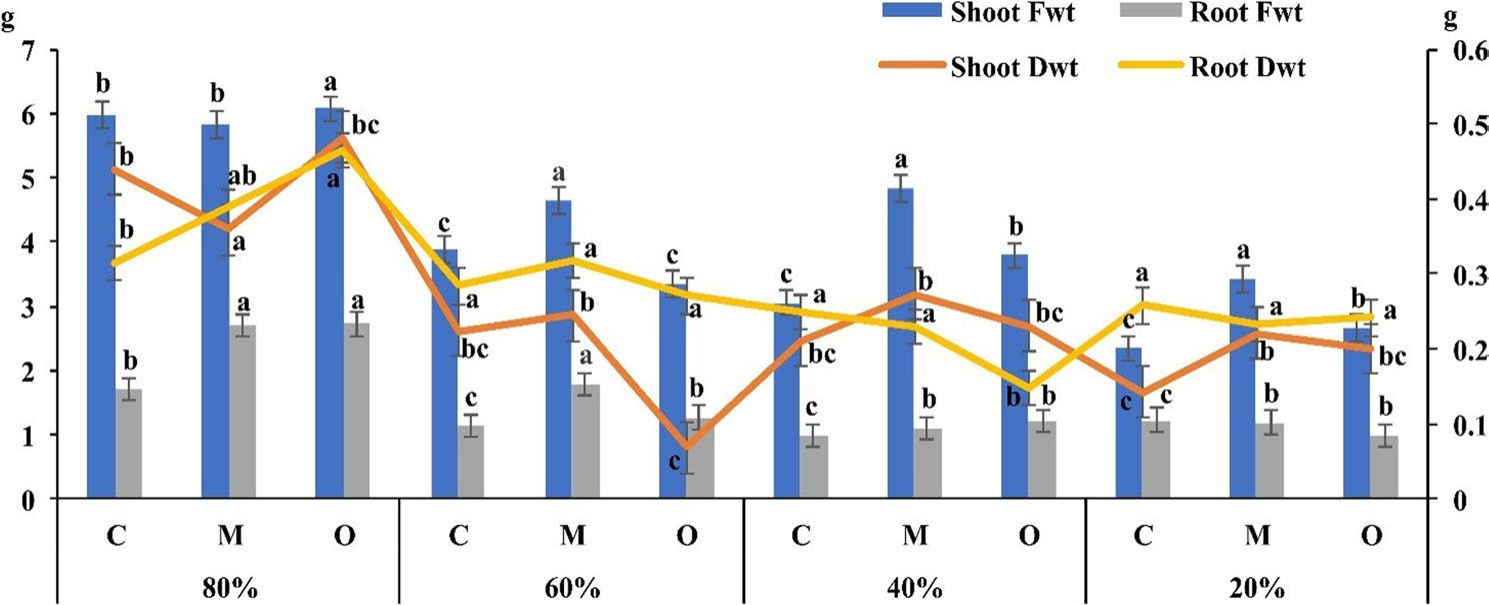
Effect of *Musa paradisiaca* L. (banana) and *Opuntia ficus-indica* (L.) Mill. (prickly pear) peel powder treatments (5 g/pot) on shoot and root fresh and dry weight of *Vicia faba* L. (faba bean) under different irrigation levels (20%, 40%, 60%, and 80% field capacity). Data are presented as clustered columns (primary axis) and lines (secondary axis). Values are expressed as mean ± standard error of three biological replicates (*n* = 3). Different letters above bars indicate significant differences according to Tukey’s HSD test at *P* ≤ 0.01

The effect of *Opuntia ficus-indica* (prickly pear) and *Musa paradisiaca* (banana) peel powder treatments on shoot and root fresh and dry weight of *V. faba* (faba bean) was evaluated under different irrigation levels (20%, 40%, 60%, and 80% field capacity) (Fig. 3). The results demonstrated significant variations in both shoot and root fresh and dry weights across treatments and irrigation levels. Plants treated with banana peel powder exhibited the greatest improvements in biomass accumulation compared to the control, followed closely by prickly pear treatment. Specifically, under moderate water availability (40% FC), shoot fresh weight increased by 58.1% relative to the control. Two-way ANOVA indicated that field capacity had a highly significant effect on all growth parameters (F = 15.91–32.7, *P* ≤ 0.01), while the treatment effect was significant for shoot fresh and dry weight as well as root fresh weight (F = 12.3–14.2, *P* ≤ 0.01), but not for root dry weight alone (F = 0.22, *P* = 0.804). The interaction between treatment and field capacity was also significant for all parameters (F = 2.6–4.6, *P* ≤ 0.01), suggesting that the efficacy of the peel powder treatments depended on the irrigation level.

**Fig. 4:**
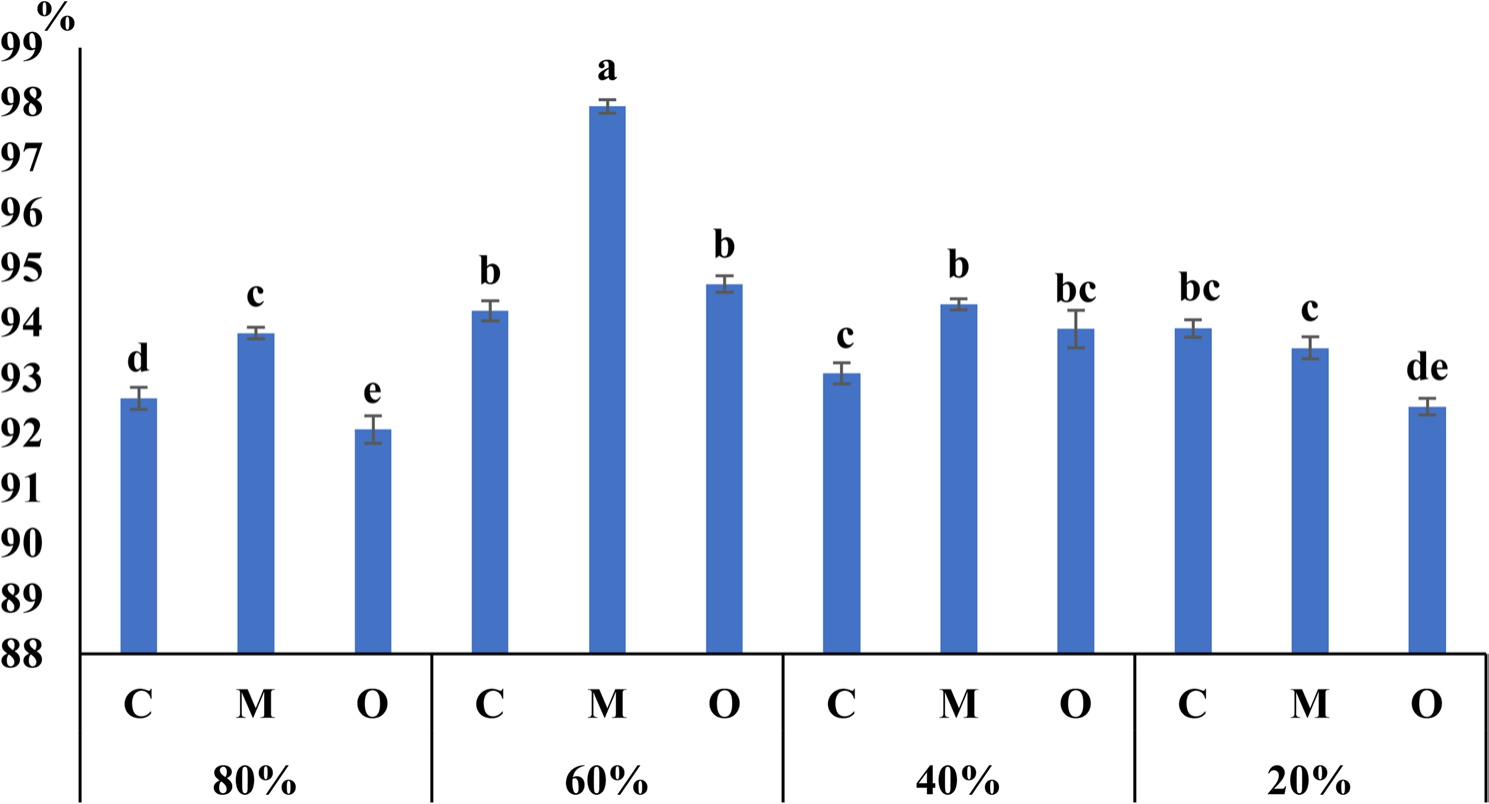
Effect of *Musa paradisiaca* L. (banana) and *Opuntia ficus-indica* (L.) Mill. (prickly pear) peel powder treatments (5 g/pot) on water content of *Vicia faba* L. (faba bean) under different irrigation levels (20%, 40%, 60%, and 80% field capacity) Columns with the same letters are not significantly different according to Tukey’s HSD test at *P* ≤ 0.01. Values are expressed as mean ± standard error of three biological replicates (*n* = 3)

The effect of peel powder applications on leaf water content of *V. faba* was significantly influenced by both irrigation level and treatment (Fig. 4). Water content varied markedly across field capacities (20%, 40%, 60%, and 80% FC), indicating a strong interaction between water availability and biostimulant application. Plants treated with *Musa paradisiaca* (banana) peel powder exhibited consistently higher water content compared with the untreated control across most irrigation levels. The most pronounced improvement was observed under moderate irrigation (60% FC), where banana peel treatment increased water content by 5.7% relative to the control. In contrast, lower irrigation levels (20% FC) resulted in reduced water content regardless of treatment, although treated plants maintained relatively higher hydration than untreated ones. These findings suggest that banana peel powder enhances plant water status, particularly under optimal or moderately reduced irrigation conditions, thereby contributing to improved physiological performance under water-limited environments.

### Effect of natural peel treatments on leaf ultrastructure of *Vicia faba*

**Fig. 5:**
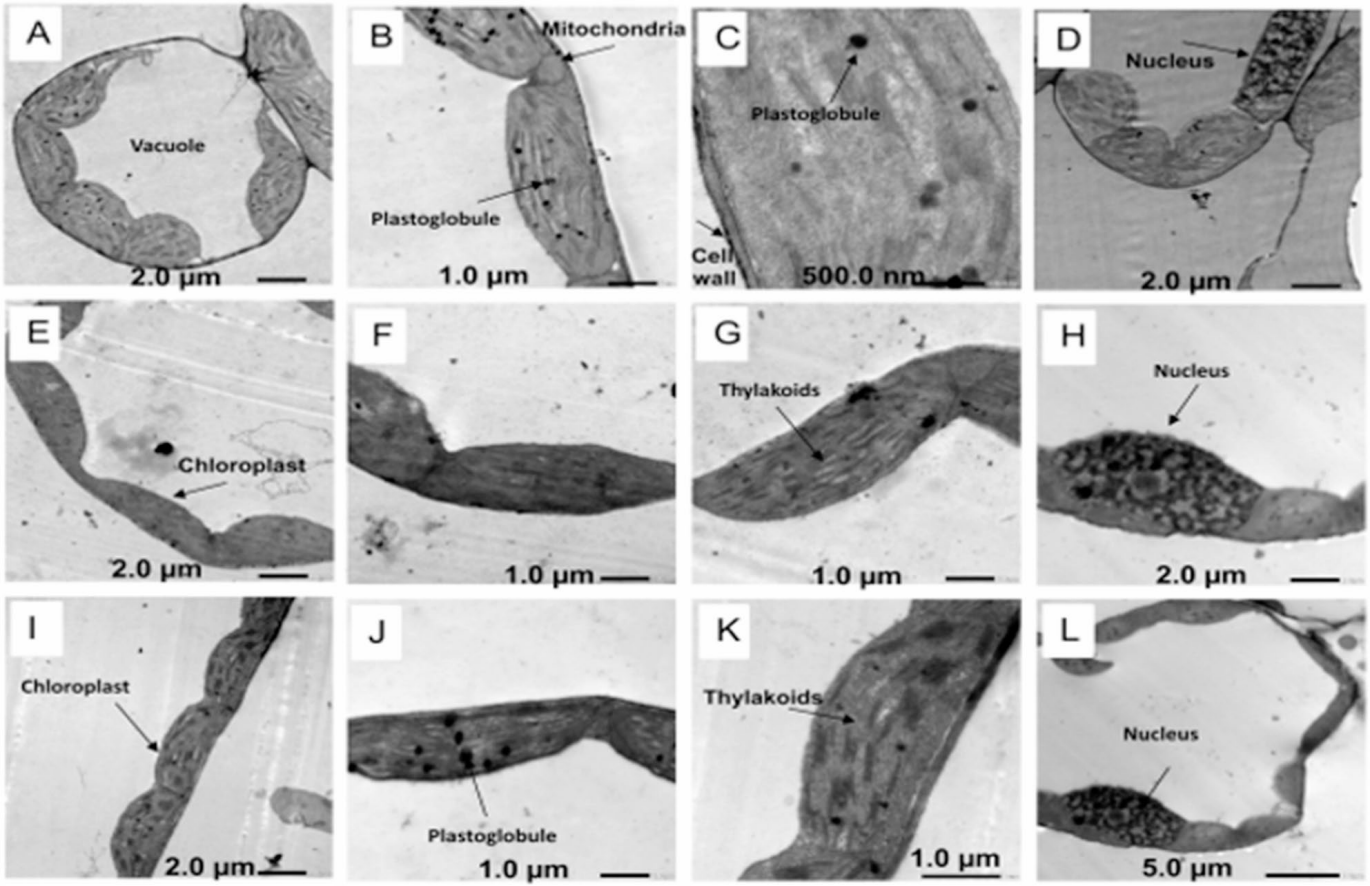
Transmission electron microscope (TEM) micrographs of *Vicia faba* leaves under control and peel powder treatments at 80% field capacity. Treatments: control (C80, **A**–**D**), banana peel powder (**E**–**H**), and prickly pear peel powder (**I**–**L**)

**Fig. 6:**
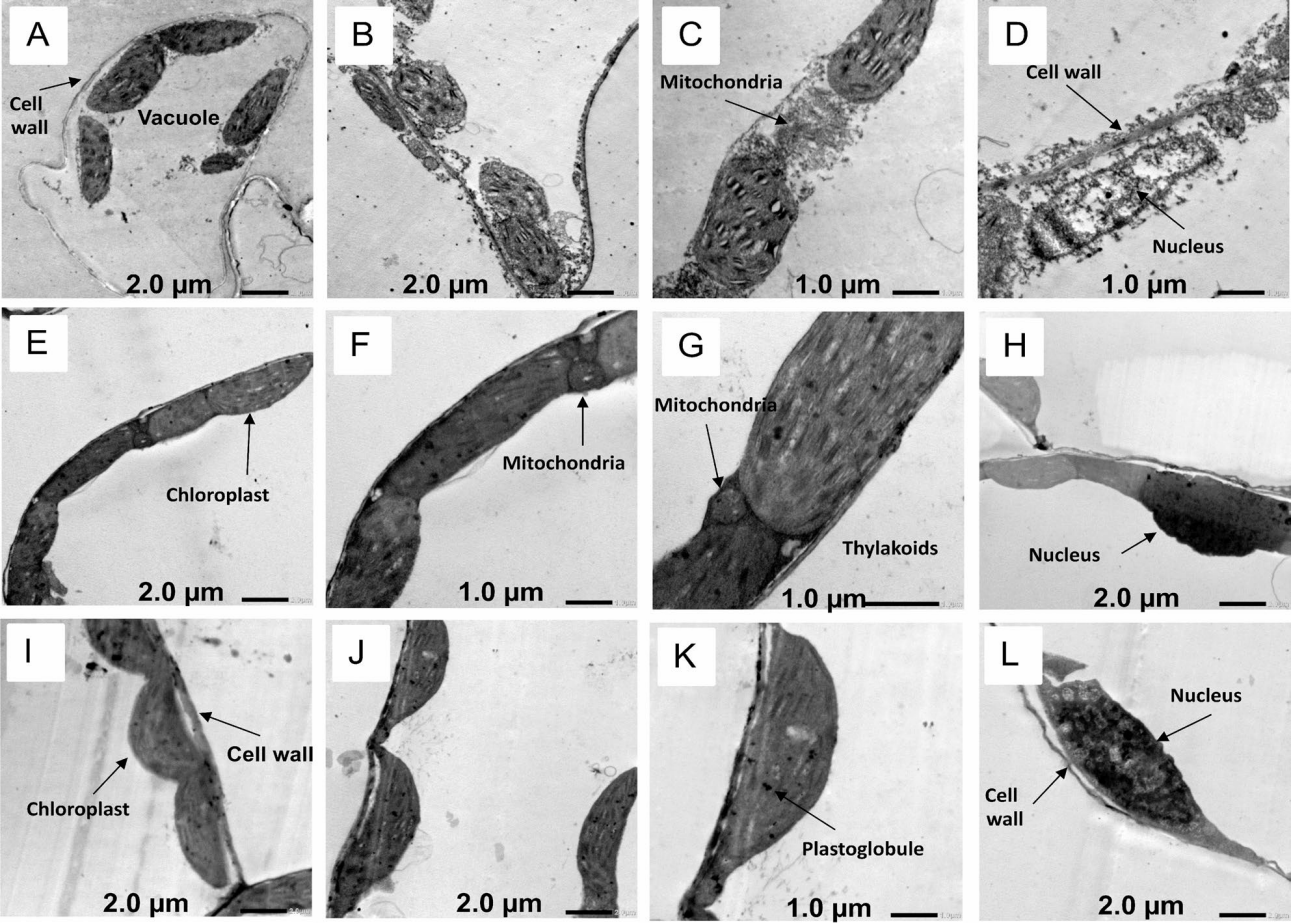
Transmission electron microscope (TEM) micrographs of *Vicia faba* leaves under control and peel powder treatments at 40% field capacity drought stress. Treatments: control (C40, **A**–**D**), banana peel powder (**E**–**H**), and prickly pear peel powder (**I**–**L**)

Transmission electron microscopy (TEM) micrographs of *V. faba* leaves are presented in Fig. 5. Leaves from control plants at 80% field capacity (C80, M80, and O80) showed well-defined cell walls, continuous cell membranes, normal chloroplasts, and intact thylakoids (Fig. 5B, G, K). No obvious changes were observed in the nucleus under these conditions. In contrast, Treatment with banana peel powder (M: *Musa paradisiaca*) and prickly pear peel powder (O: *Opuntia ficus-indica*) under drought stress (40% field capacity) improved cellular ultrastructure compared to drought-stressed control (C40). Specifically, TEM panels E-H and I-L represent the M and O treatment respectively with banana peel treatment showing more pronounced recovery (Fig. 6).

### RNA extraction, c-DNA synthesis, and qRT-PCR analysis

**Fig. 7:**
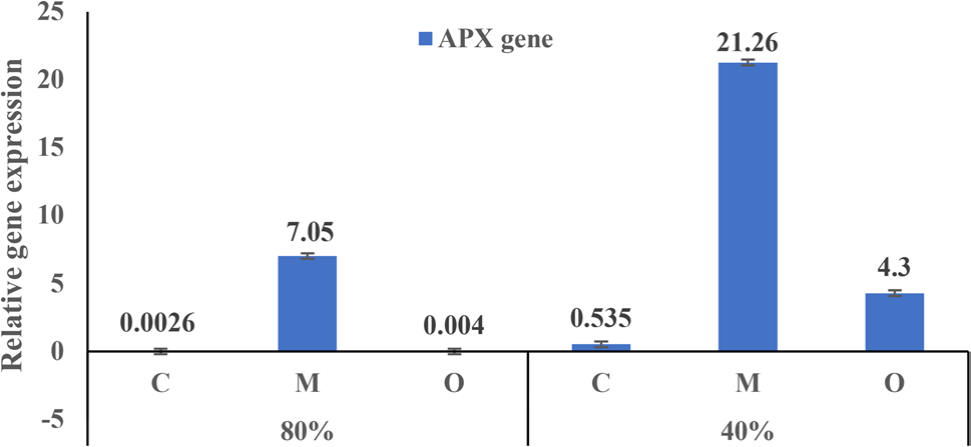
Relative expression of the APX gene in *Vicia faba* leaves under control (80% FC) and drought stress (40% FC) conditions, with the application of banana peel powder or prickly pear peel powder (5 g/pot), compared with the respective controls. Data are presented as mean ± SD of three biological replicates (*n* = 3)

The expression of the APX gene in *V. faba* leaves was significantly upregulated in all treatments compared to the control at 80% field capacity (Fig. 7). Furthermore, under normal irrigation, plants treated with *Musa paradisiaca* peel powder exhibited higher APX expression than those treated with *Opuntia ficus-indica* peel powder. Notably, under moderate drought stress (40% FC), APX expression reached a maximum level (+ 21.26) in plants treated with banana peel powder (5 g/pot).

**Fig. 8:**
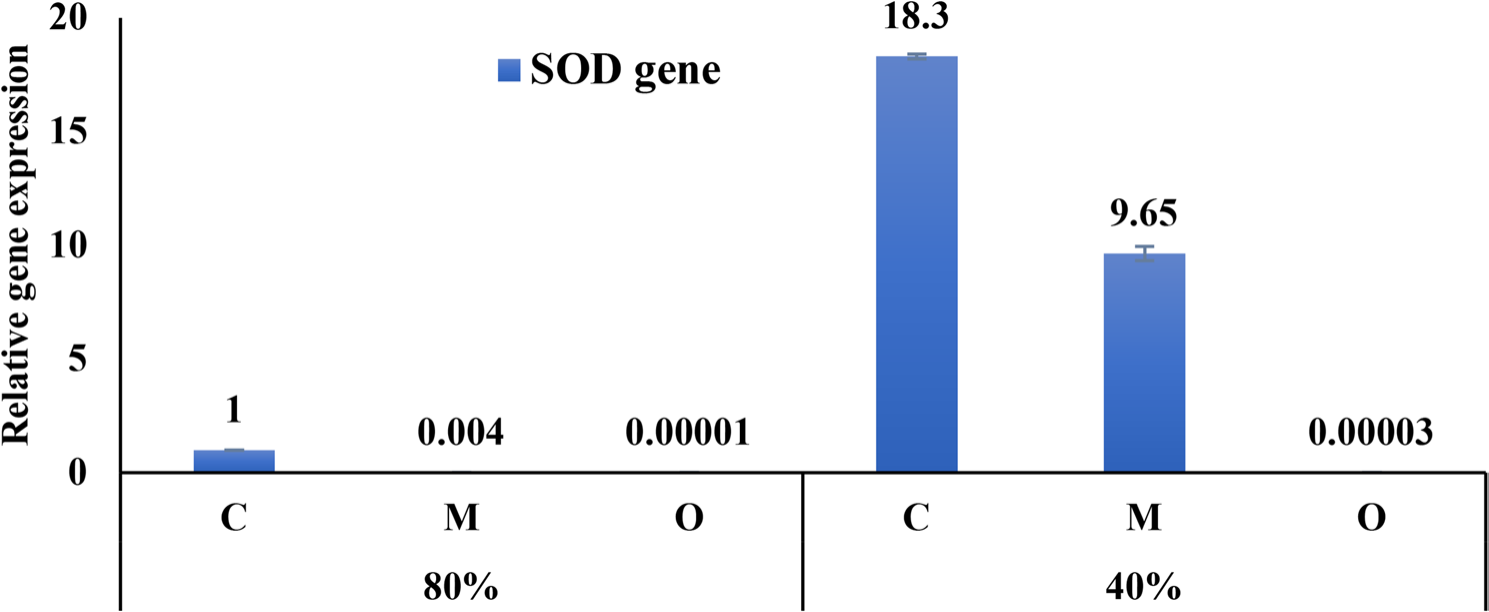
Relative expression of the SOD gene in *Vicia faba* leaves under control (80% FC) and drought stress (40% FC) conditions, with the application of banana peel powder or prickly pear peel powder (5 g/pot), compared with the respective controls. Data are presented as mean ± SD of three biological replicates (*n* = 3)

In bean leaves, the expression of SOD was reduced in the majority of treatments as compared to control (80%). The SOD gene exhibited an increase in expression in plants exposed to moderate drought stress (D40%) and in those treated with *Musa paradisiaca* peel powder under drought stress (M+D40%) as shown in Fig. 8.

**Fig. 9:**
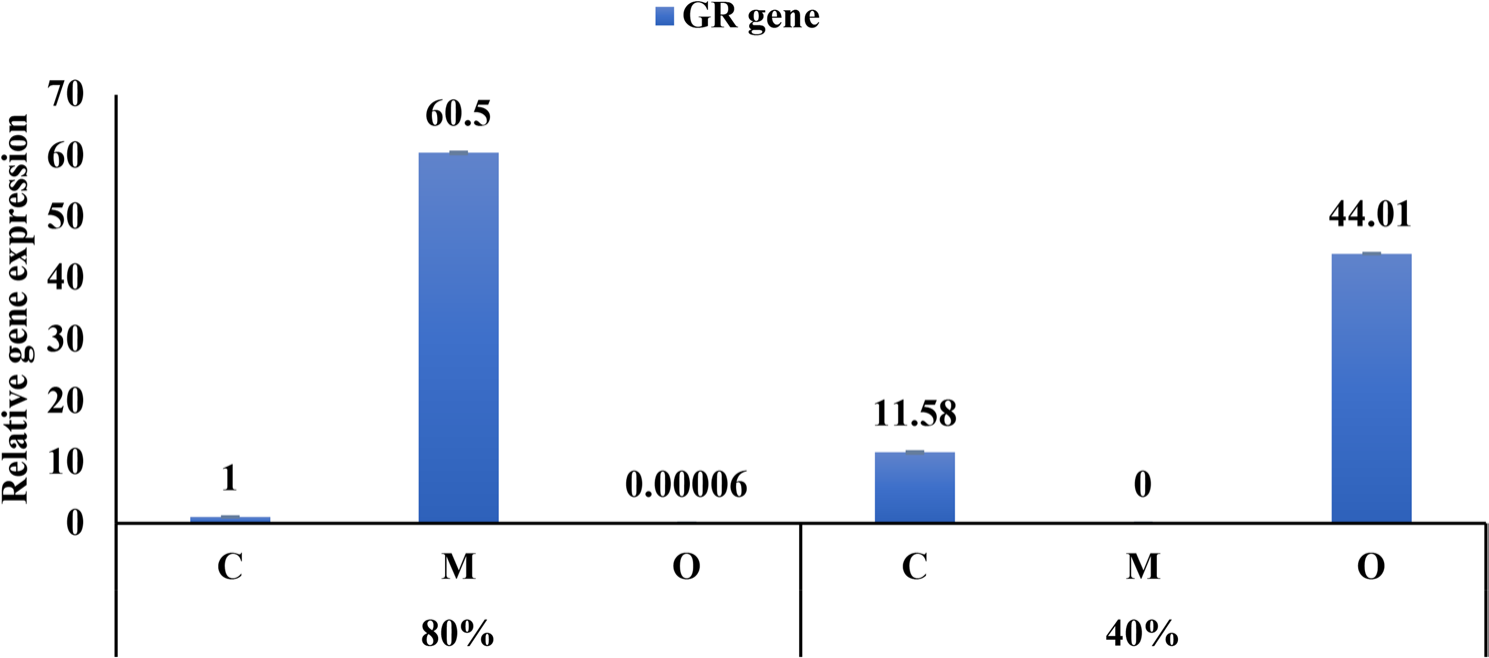
Relative expression of the GR gene in *Vicia faba* leaves under control (80% FC) and drought stress (40% FC) conditions, with the application of banana peel powder or prickly pear peel powder (5 g/pot), compared with the respective controls. Data are presented as mean ± SD of three biological replicates (*n* = 3)

The expression of GR was up-regulated in plant treated with *Musa paradisiaca* peels powder under normal condition (80%). Conversely, the expression of GR was down- regulated in plants treated with *Opuntia ficus-indica* under normal condition (80%). In contrast, plant treated with *Musa paradisiaca* peels powder under moderate drought stress 40% showed down regulation of GR expression. The expression of GR was upregulated in plants treated with *Musa paradisiaca* peel powder under well-watered conditions, whereas in plants treated with *Opuntia ficus-indica* peel powder, GR expression was higher under moderate drought stress (Fig. 9).

## Discussion

The biochemical profiles of banana (*Musa paradisiaca*) and prickly pear (*Opuntia ficus‑indica*) peels are rich in valuable bioactive compounds, supporting their potential application as natural biostimulants. Fruit peels generally contain significant amounts of soluble sugars and proteins, which can serve as energy and nutrient sources, thereby enhancing soil microbial activity and plant growth when applied to crops. This aligns with previous reports highlighting the presence of carbohydrates and proteinaceous components in peel flours used in food products, indicating their nutrient‑rich nature [48]. In addition to macronutrients, both peels are recognized for their phenolic and flavonoid compounds, which contribute considerably to antioxidant capacity. Phenolic and flavonoid constituents are widely documented as effective antioxidants, capable of mitigating oxidative stress in biological systems and potentially improving plant resilience under stress conditions [49].

The present study demonstrated that the application of banana and prickly pear peel powders significantly improved the growth performance and drought tolerance of faba bean plants, with banana peel generally having a more pronounced impact. Two-way ANOVA showed that both treatment and field capacity significantly affected shoot and root lengths, indicating that the effect of treatments depended on soil moisture levels. The prominent effect observed with banana peel compared to prickly pear peel can be attributed to its higher concentration of potassium, amino acids, and soluble sugars such as glucose, which collectively contribute to lowering the cellular osmotic potential. This reduction aids to water absorption and maintaining cell turgor under water stress conditions [50, 51]. In addition to their nutritional role, these plant-derived residues may act as sources of bioactive molecules, including plant hormones and phenolic compounds. These compounds regulate growth and activate defense mechanisms in response to stress, for example by inducing protective proteins such as dehydrins. This mechanism correspond with the demonstrated effects of banana and prickly pear peels in mitigating drought stress [4].

These findings are in agreement with previous reports highlighting the role of organic biostimulants and plant-derived residues in enhancing plant growth under water-limited conditions through improved nutrient supply, osmotic adjustment, and activation of antioxidant defenses [52, 53]. In the current experiment, shoot and root lengths, as well as fresh and dry biomass, were notably enhanced under both optimal and stress conditions, particularly with banana peel treatments. The results indicate that soil moisture is the main driver of both shoot and root growth, with high FC promoting biomass accumulation. Priming treatments enhanced growth under adequate moisture but had limited effect under water stress (40–20% FC), consistent with the significant FC and Treatment interactions observed. Similar findings were reported by [14], who demonstrated that banana residues improved growth traits of legumes under drought stress by enriching the soil with potassium, calcium, and bioactive compounds that stimulate root elongation and enhance water uptake. A recent review on banana peel valorization highlighted that composites of banana and orange peels, as well as dried banana peels, significantly improved plant growth parameters [39]. Water content was also significantly improved by peel treatments, especially at 60% field capacity, suggesting that such natural amendments help plants maintain better hydration status during stress. Organic amendments enhanced the water status and membrane stability in maize under drought conditions, increased photosynthesis pigments and relative water content, improved leaf gas exchange attributes and enhanced activities of antioxidant enzymes [54].

The observed growth-promoting and drought‑mitigating effects of banana and prickly pear peel powders may be linked to their biochemical composition, including soluble sugars, soluble proteins, and phenolic compounds. Banana peels have been reported to contain considerable levels of carbohydrates (including soluble sugars), proteins, and phenolic constituents such as flavonoids and hydroxycinnamic acids, which are known for their antioxidant capacity and ability to scavenge reactive oxygen species (ROS) under stress conditions. For example, Rawat et al. [55] found that banana peel contains significant amounts of phenolic compounds, including flavonol glycosides such as rutin, contributing to antioxidant potential. Similarly, prickly pear (*Opuntia ficus‑indica*) peels have been shown to be rich in phenolic acids and flavonoids, including gallic, coumaric, and ferulic acids, which are associated with antioxidant activity and protective effects in plant tissues [49]. These bioactive constituents may enhance plant metabolism and support antioxidant defense systems, contributing to improved tolerance to drought stress observed in *V. faba* following peel powder treatments. Collectively, these biochemical constituents may contribute to drought tolerance through osmotic adjustment, stabilization of cellular membranes, and regulation of stress-responsive pathways, thereby improving plant performance under water-limited conditions.

Drought stress is known to reduce photosynthetic efficiency because it negatively impacts thylakoid organization and chloroplast structure [56, 57]. Moreover, transmission electron microscopy revealed that untreated drought-stressed leaves exhibited severe ultrastructural alterations, including disrupted chloroplasts and thylakoids, whereas peel treatments, particularly banana, preserved cellular integrity and maintained well-defined nuclei. Comparable results were reported by [58], who demonstrated that exogenous treatments effectively protected wheat leaves against drought-induced injuries by maintaining chloroplast ultrastructure, stabilizing thylakoid membranes, and reducing membrane lipid peroxidation.

At the molecular level, the expression analysis confirmed that antioxidant-related genes were responsive to both peel treatments and drought conditions. The transcription studies in plants have long shown the correlation between environmental stresses such as salinity and drought and gene expression, particularly for antioxidant enzymes [59]. In the present study, APX expression was considerably increased in all treatments compared with the control, with the highest level observed in banana-treated plants under moderate drought stress. These results are consistent with findings by [45, 60, 61], who reported increased APX expression under drought in chickpea and Kentucky bluegrass, respectively. Similar patterns were reported under salinity stress in rice, where [62] observed upregulation of APX isoforms. APX is widely recognized as a vital enzyme in the ROS detoxification system, maintaining cellular redox balance through the AsA-GSH cycle [63]. The higher APX transcript levels under Musa treatments suggest a potential role of banana peel-derived compounds in ROS-scavenging mechanisms, which might contribute to oxidative stress mitigation, although enzymatic or oxidative stress measurements were not assessed.

The SOD gene exhibited a mixed pattern, being reduced in most treatments compared with control, but induced under moderate drought and in banana peel-treated plants at 40% FC. Similar regulatory patterns were reported by [64, 65], who showed that overexpression of cytosolic Cu/ZnSOD in transgenic plants improved tolerance to osmotic and drought stresses by enhancing chloroplast antioxidant systems. This suggests that SOD induction may occur in a treatment- and stress-dependent manner, supporting its role as a frontline defense against superoxide radicals. The GR gene displayed oscillatory regulation depending on treatment and irrigation level. It was upregulated in banana-treated plants under normal conditions but downregulated under drought. Comparable patterns were noted by [66] in cowpea, where cytosolic GR expression was directly related to drought intensity. Similarly [67], found that GR remained stable under moderate stress in tolerant cowpea, with upregulation occurring during recovery, suggesting acclimation. These results indicate that GR expression in faba bean may reflect a fine-tuned balance between constitutive defense and stress-induced recovery, with banana peel treatment exerting condition-dependent regulation.

## Conclusion

In conclusion, these results indicate that natural peel powders, particularly those derived from banana, enhance plant performance during drought by improving growth, maintaining cellular ultrastructure, and modulating the expression of antioxidant genes. The integration of physiological, ultra-structural, and molecular evidence supports the notion that biostimulants derived from peels activate various protective pathways, which may contribute to reduced oxidative damage and sustaining plant metabolism during stress. The stronger effect of banana peel may be related to its higher nutrient and phytochemical content, which could influence antioxidant-related pathways more prominently than prickly pear. Overall, these findings support a possible role of antioxidant enzymes such as APX, SOD, and GR in drought tolerance, as suggested by their gene expression patterns. and also highlight the potential of agricultural byproducts as sustainable tools for enhancing crop resilience under climate change-driven water scarcity.

## Data Availability

All data generated or analyzed during this study are included in the published article.

## Abbreviations

ANOVA: Analysis of Variance
APX: Ascorbate Peroxidase
AsA–GSH cycle: Ascorbate–Glutathione Cycle
CAT: Catalase
Cdna: Complementary Deoxyribonucleic Acid
Ct: Cycle Threshold
DHAR: Dehydroascorbate Reductase
DNA: Deoxyribonucleic Acid
FC: Field Capacity
GR: Glutathione Reductase
mRNA: Messenger Ribonucleic Acid
qRT-PCR: Quantitative Real-Time Polymerase Chain Reaction
ROS: Reactive Oxygen Species
RTL: Relative Transcript Level
SOD: Superoxide Dismutase
TEM: Transmission Electron Microscopy
TRIzol: Tri Reagent for RNA Isolation
